# Mitochondrial metabolism and targeted treatment strategies in ischemic-induced acute kidney injury

**DOI:** 10.1038/s41420-024-01843-5

**Published:** 2024-02-10

**Authors:** Yongming Chen, Zixian Li, Hongyong Zhang, Huixia Chen, Junfeng Hao, Huafeng Liu, Xiaoyu Li

**Affiliations:** 1 Institute of Nephrology, and Guangdong Provincial Key Laboratory of Autophagy and Major Chronic Non-Communicable Diseases, Affiliated Hospital of Guangdong Medical University, Zhanjiang, 524001 China; 2 Zhanjiang Institute of Clinical Medicine, Central People’s Hospital of Zhanjiang, Guangdong Medical University Zhan-jiang Central Hospital, Zhanjiang, 524001 China

**Keywords:** Mitophagy, Cell death, Predictive markers, Acute kidney injury

## Abstract

Ischemia-reperfusion injury (IRI) is a common cause of acute kidney injury (AKI). The kidney is susceptible to IRI under several clinical conditions, including hypotension, sepsis, and surgical procedures, such as partial nephrectomy and kidney transplantation. Extensive research has been conducted on the mechanism and intervention strategies of renal IRI in past decades; however, the complex pathophysiology of IRI-induced AKI (IRI-AKI) is not fully understood, and there remains a lack of effective treatments for AKI. Renal IRI involves several processes, including reactive oxygen species (ROS) production, inflammation, and apoptosis. Mitochondria, the centers of energy metabolism, are increasingly recognized as substantial contributors to the early phases of IRI. Multiple mitochondrial lesions have been observed in the renal tubular epithelial cells (TECs) of IRI-AKI mice, and damaged or dysfunctional mitochondria are toxic to the cells because they produce ROS and release cell death factors, resulting in TEC apoptosis. In this review, we summarize the recent advances in the mitochondrial pathology in ischemic AKI and highlight promising therapeutic approaches targeting mitochondrial dysfunction to prevent or treat human ischemic AKI.

## Facts

1. The hallmarks of IRI-AKI are elevated levels of ROS, mitochondrial dysfunction, and inflammation.
2. Mitochondrial dysfunction is a potential mechanism underlying energy metabolism disorder.
3. Loss of transcription factor A (TFAM) caused by mtROS led to mitochondrial dysfunction in TECs.
4. Mitophagy has a protective effect on TECs during AKI.
5. Screening for effective interventions to regulate mitochondrial metabolism should be the focus of future research.

## Open questions

1. What is the specific protective mechanism of Sirt5 deficiency underlying renal tubule injury?
2. Can UMI-77 targeting mitophagy improve AKI? What is the specific mechanism?
3. Can drugs targeted to regulate mitochondrial metabolism treat CKD?

## Introduction

Acute kidney injury (AKI) is a common clinical condition comprising complex pathophysiological processes. AKI is associated with high morbidity and mortality and lacks clear and effective treatments. A meta-analysis of the global incidence of AKI showed that the combined incidence of AKI was 23.2% when the KDIGO-equivalent AKI definition was used across 154 studies (130 adult and 24 child studies). The all-cause mortality associated with AKI was 23.0% in 110 studies (99 adult and 11 child studies) that used KDIGO-equivalent AKI to define and assess mortality. AKI affects up to 20% of hospitalized patients [1]. A British study showed that the total annual medical expenses related to inpatients with AKI are as high as 1.02 billion pounds, indicating that AKI exerts a great burden on the medical and social economy [2].

The kidneys of ischemia-reperfusion injury (IRI)-AKI mice exhibit severe mitochondrial damage, as evidenced by increased mitochondrial ROS levels and decreased ATP levels in the proximal tubule cells [3, 4]. Mitochondrial lesions, such as mitochondrial swelling and fragmentation, disruption of membrane integrity, and broken or absent cristae, have been observed in renal tubules of IRI-AKI mice [4]. Healthy mitochondria generate ATP, buffer cytosolic calcium, and produce appropriate ROS [5]. Furthermore, healthy mitochondria mediate energy utilization, signal transduction, and apoptosis in cells [6]. Dysfunctional mitochondria disrupt intracellular homeostasis and induce lethal injury. The accumulation of damaged mitochondria causes serious energy deficiency, impaired calcium buffering, increased ROS production, apoptosis due to pro-apoptotic factors released into the cytoplasm, and necrosis due to the opening of the mitochondrial permeability transition pore [7]. Thus, the maintenance of the number, morphology, and function of mitochondria, which act as energy powerhouses, is essential for cell health. Studies on mitochondria may provide greater insight into the mechanism underlying IRI-AKI. Furthermore, the restoration of healthy mitochondrial function and mass is critical for the recovery of kidney function.

Recent advancements regarding the role of mitochondria in IRI-AKI has led to their being used in an array of potential applications as biomarkers of kidney injury and targets for novel therapeutic strategies. In this review, we describe mitochondrial metabolic dysregulation in ischemic AKI and discuss how this knowledge may guide the development of novel therapies for ischemic AKI.

## Abnormal morphology and function of mitochondria during ischemia-induced AKI

Mitochondria constitute the main energy producers in cells, and the kidneys are second only to the heart in terms of mitochondrial number and oxygen consumption. The primary cause of acute kidney disease is thought to be related to mitochondrial damage and impaired function [8–10]. In the physiological state, mitochondria exhibit normal tubular morphology [11]. Functionally, mitochondria produce ATP via oxidative phosphorylation during cellular energy metabolism [12]. In IRI-AKI, mitochondria become smaller and more rounded in atrophied tubules [13]. However, alterations in energy metabolism in TECs of IRI-AKI mice can lead to changes in the balance between mitochondrial and peroxisomal fatty acid oxidation (FAO), thereby aggravating AKI [14]. Additionally, mitochondrial damage increases ROS production and induces mitophagy. The loss of PINK1, PARK2, BINP3, and other proteins can block mitophagy and aggravate ROS accumulation, thereby causing mitochondrial accumulation and TEC damage, ultimately aggravating AKI [15, 16].

### Mitochondrial pathology in atrophic tubules following IRI

In human kidneys, mitochondria in TECs of patients with AKI are swollen and fragmented [17]. In a Sprague-Dawley rat model subjected to bilateral renal ischemia for 45 min, endothelial cells, podocytes, and TECs showed significant mitochondrial damage after nine months of ischemia [18]. After the death of proximal tubule cells due to AKI, the surviving epithelial cells dedifferentiate, migrate, and proliferate [19]. Recovery of normal structure and function occurs through redifferentiation of the reconstituted epithelium. However, to varying degrees, proximal tubule cells proliferating after AKI fail to re-differentiate, undergo premature growth arrest, become atrophic, and exhibit a flat simplified cytoplasm. In rat kidneys, tubules undergoing atrophy following IRI and abnormal recovery show greatly reduced mitochondrial numbers, as well as smaller sizes, with round profiles. Moreover, atrophic tubules exhibit large complex autophagolysosomes containing degenerate mitochondria [13].

### Mitochondrial energy metabolism changes in ischemia-induced AKI

**Fig. 1:**
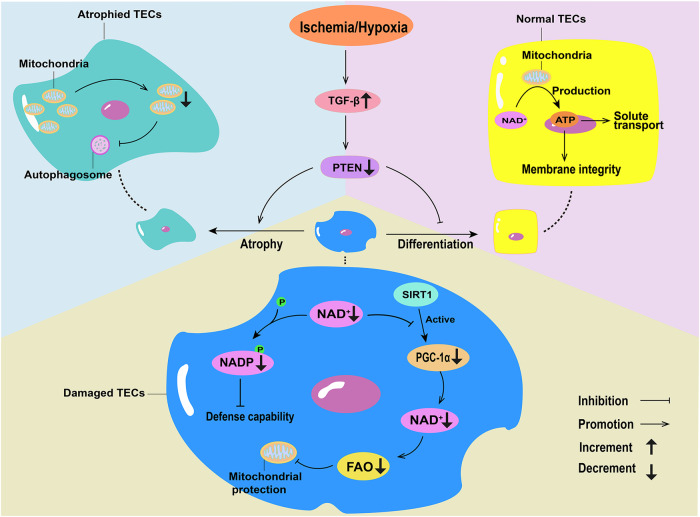
Changes in mitochondrial energy metabolism in ischemia-induced acute kidney injury (AKI). Hypoxia can trigger TGF-β, and the increase in TGF-β leads to PTEN defects in renal tubules, inducing tubule atrophy, reducing mitochondrial mass, and causing abnormal autophagy. In healthy tubular epithelial cells (TECs), NAD^+^ produces ATP through the mitochondria, supplying solute transport and maintaining membrane integrity. In normal TECs, SIRT1 activates PGC1α through the de novo pathway, and promotes the production of NAD^+^, thereby enhancing fatty acid oxidation (FAO) and mitochondrial protection. In damaged TECs, the consumption of NAD^+^ inhibits PGC1α, reduces the production of NAD^+^, decreases FAO, and weakens the protective effect on mitochondria. The depletion of NAD^+^ inhibits the phosphorylation of NADP, and decreases the defense ability of TECs against ischemia-reperfusion injury-induced oxidative stress.

Under normal conditions, TECs primarily depend on FAO as their energy source [20]. Following IRI in a Sprague-Dawley rat model, tubules that failed to re-differentiate continued to exhibit increased glycolysis and higher glycolytic enzyme expression, an alteration accompanied by increased expression of hypoxia markers. In atrophic tubules of IRI rat kidneys, the levels of carbonic anhydrase 9 (CA9), a hypoxic marker directly induced by HIF-1α, significantly increased. In addition, hypoxia can trigger TGF-β, which is involved in the pathogenesis of ischemic tubular atrophy. TGF-β signal in the rat kidney of IRI continuously increased, resulting in PTEN defects in renal tubules, which interfered with the differentiation of damaged tubules, thereby inducing tubule atrophy. When tubule atrophy occurs, mitochondrial mass markedly decreases and autophagy becomes abnormal (Fig. 1) [13, 21, 22]. Thus, renal tubule atrophy leads to increased CA9 expression, which further aggravates tubule atrophy, leading to abnormal mitochondrial metabolism. Potter et al. found that mitochondrial division and autophagy are enhanced after CA9 inhibition in malignant mesothelioma cells [23]. Therefore, the targeted regulation of CA9 expression may regulate mitochondrial energy metabolism, thereby improving IRI-AKI. In healthy mitochondria, electron transport along the electron transport chain is coupled with oxidative phosphorylation to produce ATP. In the ischemic state of the mitochondria, the activity of various complexes in the electron transport chain is reduced, leading to electron leakage and reducing oxygen formation of superoxide radicals when oxygen is available during reperfusion, ultimately reducing the amount of ATP available [24]. Nicotinamide adenine dinucleotide (NAD) is required for glycolysis, FAO, and the tricarboxylic acid cycle, leading to ATP generation via the electron transport chain in mitochondria. In healthy renal TECs, ATP provides the energy required for key functions, such as solute transport and the maintenance of membrane integrity. NAD in the mitochondria is the rate-limiting step in mitochondrial energy production [25]. Rats subjected to renal IRI show accelerated NAD consumption. Therefore, NAD cannot interact well with Sirtuins and PPARγ co-activator 1α (PGC1α) to alleviate renal tubular injury. Sirtuin 1 (SIRT1) activates PGC1α through NAD^+^-dependent deacetylation, and PGC1α promotes the biosynthesis of NAD^+^ via the de novo pathway by synergistically upregulating the expression of genes encoding the NAD^+^ pathway. Increased NAD^+^ levels can enhance FAO and active a broad cellular regulatory signaling network to protect against oxidative metabolism and mitochondrial damage, consequently protecting against renal tubular injury. NAD consumption may also reduce phosphorylation of NAD to NADP, which may weaken the defense of TECs against oxidative stress induced by IRI (Fig. 1) [26, 27].

Additionally, Sirt5 regulates the balance between mitochondrial and peroxisomal FAO in proximal TECs to protect against AKI. Following ischemia-induced AKI, Sirt5 deficiency regulates proximal TECs to increase peroxisomal FAO when compared with mitochondrial FAO, thereby reducing oxygen demand, alleviating oxidative stress, significantly improving renal function and less tissue damage [14]. Mou, Luohe et al., found that compound 58(IC50 = 0.31 μM), an inhibitor of SIRT-5, regulates protein succinylation and proinflammatory cytokines in the kidneys of mice with septic AKI, thereby showing renal protection in vivo [28]. However, the specific protective mechanism of Sirt5 deficiency against IR-induced renal tubular injury remains unclear and requires further investigation.

During ischemia-induced AKI, the mitochondrial energy metabolic balance in TECs is altered. Thus, the inhibition of mitochondrial mass loss due to tubule atrophy, mitochondrial energy deficit due to NAD depletion, and mitochondrial FAO levels can improve renal function, thereby rendering mitochondria potential therapeutic targets.

### Mitophagy in ischemia-induced AKI

Mitophagy is a type of selective autophagy that tags damaged or dysfunctional mitochondria for autophagic recognition and degradation. As a critical component of mitochondrial quality control, mitophagy can prevent excessive ROS production and release of mitochondrial pro-apoptotic factors, and inhibit pathological inflammatory responses [29, 30]. Two mitophagy priming mechanisms have been proposed that rely on the PTEN-induced kinase 1 (PINK1)-parkin RBR E3 ubiquitin-protein ligase (PARK2/PRKN) pathway [15], and mitophagy receptors, BCL2-interacting protein 3 (BNIP3L/NIX), BCL2-interacting protein 3 (BNIP3), or FUN14 domain-containing 1 [16, 31].

**Fig. 2:**
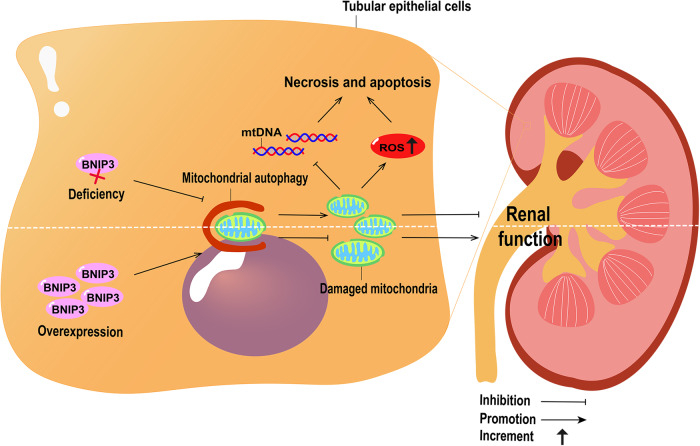
Mitophagy in ischemia-induced acute kidney injury (AKI). Mitochondrial damage increases reactive oxygen species (ROS) production, triggers mitochondrial DNA (mtDNA) damage, damages cells, and eventually leads to cell necrosis and apoptosis. BNIP3 deficiency reduces mitophagy, causes the accumulation of damaged mitochondria, and aggravates kidney injury. Overexpression of BNIP3 can increase mitophagy, reduce damaged mitochondria, and improve kidney injury.

Renal IRI induces PINK1-PARK2-mediated mitophagy in TECs in mice. Mice deficient in Pink1 or Park2 alone or in combination show aggravated mitochondrial damage and enhanced inflammatory cell infiltration during renal IRI [15]. In damaged mitochondria, PINK1 accumulates in the mitochondrial outer membrane, which binds to the translocase of the outer mitochondrial membrane, and is activated by autophosphorylation. Activated PINK1 then phosphorylates ubiquitin, triggering PRKN recruitment to the mitochondria and activating their E3 ligase activity, ultimately ubiquitinating the mitochondrial substrate and initiating mitophagy [32]. BNIP3 deficiency reduces renal IRI-induced mitophagy in mice, resulting in the accumulation of damaged mitochondria and TECs, and exacerbation of renal injury (Fig. 2) [16]. However, BNIP3 overexpression improves kidney damage and increases mitophagy in tubular HIF-1α knockout mice with IRI (Fig. 2) [33]. Li et al. found that the inhibition of dynamin-related protein 1 (Drp1) phosphorylation by mitochondrial division inhibitor-1 (Mdivi-1) significantly inhibits renal IRI-induced mitophagy in rats without affecting general autophagy, and confirmed that the downregulation of mitophagy significantly aggravated cell apoptosis and IRI-induced renal dysfunction [34].

These studies suggest that downregulation of mitophagy can lead to the accumulation of damaged and dysfunctional mitochondria, aggravating TEC injury, whereas upregulation of mitophagy can alleviate IR-induced AKI.

### ROS metabolism in ischemia-induced AKI

The destructive role played by ROS in IRI is widely recognized. As a result of the disruption of the electron transport chain caused by ischemia, ROS are produced by several sources, including NADPH oxidase, xanthine oxidase-hypoxanthine, nitric oxide synthase, and parenchymal mitochondria [35]. Renal mitochondrial injury following ischemia is associated with cellular damage resulting from ROS generation. Mitochondrial DNA (mtDNA) damage is triggered by the loss of mitochondrial membrane potential and instability of the electron transport chain, which directly damages cellular components, resulting in necrosis and apoptosis (Fig. 2) [4]. Mitochondria constitute the main source of intracellular ROS and contain numerous enzymes that convert molecular oxygen into superoxides or their derivatives, such as hydrogen peroxide [36]. In an in vitro model of IRI-AKI, increased ROS production caused direct oxidative damage to mitochondrial proteins and lipids, thereby impairing mitochondrial bioenergetics by disrupting electron transport chain function and increasing mitochondrial membrane permeability. In addition, mtROS can induce kidney damage by activating pro-inflammatory signals, such as toll-like receptors and the NLRP3 inflammasome. Elevated ROS levels are a characteristic of IRI-induced AKI [4].

**Fig. 3:**
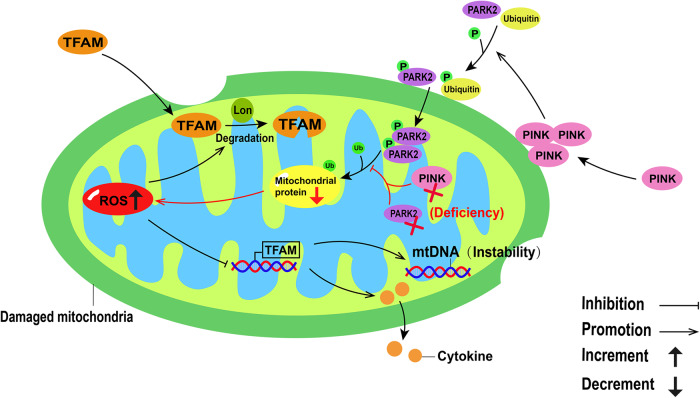
Reactive oxygen species (ROS) metabolism in damaged mitochondria. In damaged mitochondria, ROS induces mitochondrial DNA (mtDNA) instability and cytokine release by inhibiting mitochondrial transcription factor A (TFAM) transcription, while promoting Lon-mediated TFAM degradation. In damaged mitochondria, PINK1 aggregates in the mitochondrial outer membrane and phosphorylates ubiquitin and PARK2. After PARK2 aggregates in depolarized mitochondria, it ubiquitinates mitochondrial proteins. When Pink1 or Park2 is deficient, mitochondrial protein ubiquitination is inhibited and ROS production is increased.

Lon, a quality control protease, has a unique function in the mitochondrial genome to regulate mtDNA metabolism and reshape nucleoid composition. Mitochondrial TFAM is a nuclear-encoded protein synthesized in the cytoplasm, imported into the mitochondria, and removed by Lon-mediated degradation [37]. In IRI-AKI mice, mitochondrial ROS induces mtDNA instability and cytokine release by suppressing TFAM transcription and promoting Lon-mediated TFAM degradation in TECs (Fig. 3). TFAM deficiency and mtDNA damage have also been observed in the kidneys of patients with AKI [4]. In healthy mitochondria, PINK1, a serine/threonine kinase targeting mitochondria, inserts into the mitochondrial inner membrane and intermembrane space by combining with the translocator of the outer mitochondrial membrane complex, and is rapidly degraded by the stromal processing peptidase. When mitochondria are injured, PINK1 accumulates in the mitochondrial outer membrane, and phosphorylates ubiquitin and the E3 ubiquitin ligase Parkin [38]. Parkin is recruited to depolarize mitochondria and ubiquitinates several mitochondrial proteins [39]. In an in vivo model of renal IRI, Pink1 or Park2 deficiency leads to the accumulation of damaged mitochondria in renal tubular cells, decreases mitochondrial protein ubiquitination, and increases ROS production (Fig. 3) [15]. BNIP3 is a BCL-2 family protein, primarily located in the outer membrane of mitochondria. BNIP3 has been identified as a pro-apoptotic protein that sensitizes BAX (BCL2-associated X, apoptosis regulator) and BAK (BCL2 antagonist/killer 1) insertion and activation in the mitochondria, and releases pro-apoptotic factors to initiate apoptotic cascades. Alternatively, BNIP3 is also a type of mitophagy receptor, which plays a role in promoting survival under some pathological conditions. Notably, BNIP3 deficiency increases mitochondrial fragmentation, swelling, and loss of mitochondrial matrix vacuoles and cristae. Tang et al. demonstrated that renal IRI in Bnip3-KO mice caused more mitochondrial damage in renal proximal tubular cells than in wild-type mice, and that ROS levels were also significantly elevated [16].

These studies suggest that elevated ROS levels in kidneys following IRI aggravate damaged and dysfunctional mitochondria, thereby exacerbating AKI. This indicates that AKI can be ameliorated by inhibiting the increase in ROS levels in TECs following IRI.

### Disruption of mitochondrial fusion and fission in AKI

Mitochondria are highly dynamic organelles that undergo fission and fusion, which are closely related to their functions [40]. Disruption of mitochondrial dynamics has been confirmed in AKI and may ultimately lead to cell damage and death [41].

Drp1, a key protein involved in mitochondrial fission, is activated during mitochondrial dysfunction during tubule cell injury and mediates mitochondrial breakage. During mitochondrial division, cytoplasmic localized Drp1 is recruited to the division site of the mitochondrial outer membrane, where it oligomerizes, leading to assembly into helical structures to mediate membrane splice [42]. Drp1 expression increases in the renal IRI model [43]. The key mediators of mitochondrial fusion include the outer membrane fusion proteins, Mitofusin-1(Mfn1), Mitofusin-2 (Mfn2), and the inner membrane fusion protein optic atrophy 1 (Opa1) [44, 45]. In the kidneys of IR mice, Opa1 expression is significantly decreased, which leads to the obstruction of mitochondrial intima fusion and secondary mitochondrial dysfunction, and finally aggravates proximal renal tubule injury [42, 46, 47]. Notably, the kidneys of IRI mice exhibit an increase in Mfn1 expression, and a significant decrease in Mfn2 expression when compared to those of the sham group [42, 48, 49]. These results indicate that mitochondrial fusion is partly suppressed following IRI.

## Therapeutic targeting of mitochondria in ischemia-induced AKI

### Targeting mitochondrial energy metabolism

Mitochondrial energy metabolism includes pyruvate oxidation, FAO, citric acid cycle (TCA), and five oxidative phosphorylation complexes [50]. The above-mentioned imbalance in mitochondrial FAO could be used as a potential target for mitochondria-targeted therapy. FAO levels decline following renal IRI. Portilla et al. found that upregulation of the peroxisome proliferator-activated receptor-α (PPARα)-regulated FAO gene exerts an important protective effect on renal tubular cells during IRI. Mitochondrial carnitine palmitoacyltransferase 1 (CPT1) activity inhibitors can be used as direct ligands of PPARα, and this ligand-mediated activation upregulates the activities of peroxisome acyl-coA oxidase and the microsomal somatic pigment P-450, thereby increasing FAO levels and ameliorating renal tubular injury following IR [50, 51]. Hypoxic preconditioning and extracellular MSC vesicles inhibit renal fibrosis by restoring CPT A-mediated mitochondrial FAO, which can be achieved by regulating mitochondrial homeostasis [52] (Table 1).

**Table 1:** Mitochondria-targeted therapeutics.

Therapeutics	Action mechanisms	Experimental model	PMID
***Targeting mitochondrial energy metabolism***			
CPTI	Promotes FAO	IRI-AKI	10751229
AICAR	Regulates the AMPK pathway		22324445, 25607838
PDH/CS/AKGDH	Promotes TCA/oxidative phosphorylation		31900386, 34158150, 35355864
Complex I/Complex II			
Glutamine			30127395
***Targeting mitophagy***			
HIF-1α-BNIP3	Mitophagy	IRI-AKI	32829253
PINK-PRKN/PARK2			29172924
Drp1			29704468
Fundc1			31901590
UMI-77		Renal fibrosis	35524750
***Tarteting ROS***			
IDH2	Promotes ROS clearance	IRI-AKI	27821630
BI1	Inhibits ROS production		31903127
MitoQ	Inhibits ROS production		25965144
***Tarteting mitochondrial fusion and fission***			
Mdivi-1	Inhibits mitochondrial fission	IRI-AKI	29084809
P110	Inhibits mitochondrial fission		30981733, 23239023
TQ	Promotes mitochondrial fussion		21718287, 33165700
***Other therapies targeting mitochondria***			
SS-31	Increases ATP and reduces ROS	IRI-AKI	21546574
FGF2	Alleviates mitochondrial damage		32266254

The pyruvate generated by glycolysis in the TCA cycle is converted into acetyl-CoA through the action of pyruvate dehydrogenase (PDH). This acetyl-CoA is then metabolized to carbon dioxide by enzymes such as citrate synthase (CS) and alpha-ketoglutarate dehydrogenase (AKGDH) [53]. During the TCA cycle, electrons generated undergo oxidative phosphorylation to generate ATP [53]. The mitochondrial respiratory chain complex regulates mitochondrial oxidative phosphorylation, which is a crucial component of mitochondrial energy metabolism [54]. Liao et al. have shown that in renal IRI models, fluorofenidone retains the expression of PDH, CS, AKGDH, complex I and complex II in the IRI model, alleviating the damage of TCA circulation and mitochondrial respiratory chain in IRI, thereby improving mitochondrial energy metabolism [55]. This suggests that by targeting key enzymes in the TCA cycle or oxidizing related complexes in the respiratory chain, we can enhance mitochondrial energy metabolism and protect against renal IRI.

In addition, mitochondria are a major site of glutamine metabolism in various cell types [56]. Katharina et al. demonstrated that glutamine treatment significantly upregulates proteins associated with oxidative phosphorylation in mitochondria, particularly a critical subunit of NADH: ubiquinone oxidoreductase (Complex I). It has been shown that NAD^+^ synthesis damage enhances the development of AKI [57]. Notably, in a Phase I clinical trial, enhanced biosynthesis of NAD^+^ reduced the incidence of postoperative AKI in patients undergoing cardiac surgery [57]. Glutamine is involved in the regulation of oxidative stress because it is a precursor for many antioxidant molecules, such as glutathione and NAD^+^ [58–61]. Therefore, targeted upregulation of glutamine in mitochondria can increase the expression of Complex I, elevate the NAD^+^/NADH ratio, enhance the oxidative phosphorylation process, and ultimately improve mitochondrial function and mitigate kidney injury.

Nutrient-sensing pathways can directly affect mitochondrial energetics in response to external stimuli such as hypoxia, oxidative stress, and energy depletion. In particular, two signaling pathways have been explored extensively in the kidney: the mechanistic target of rapamycin [62] and AMP-activated protein kinase (AMPK) signaling pathways [63]. The AMPK activator 5-aminoimidazole-4-carboxamide-1-β-D-riboside (AICAR) prevents glomerulopathy and tubulointerstitial fibrosis in mice by stimulating FAO. AICAR also has a therapeutic effect on mouse renal IRI [41, 64].

### Targeting mitophagy

**Fig. 4:**
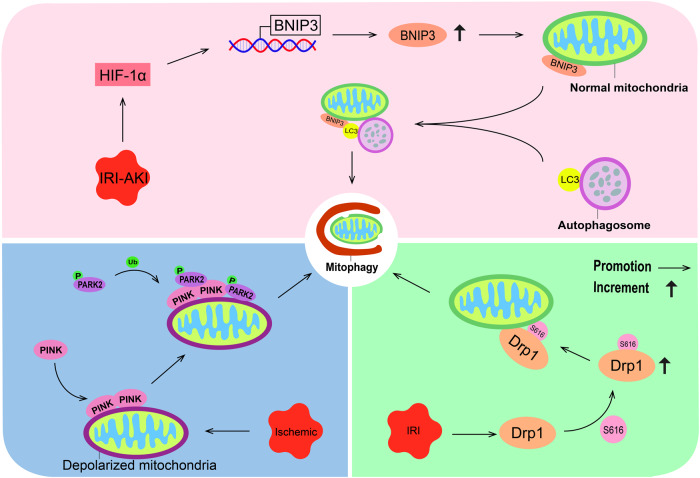
Targeting activation of mitophagy. In the IRI-AKI state, HIF-1α increases the expression of BNIP3, which localizes to the mitochondrial outer membrane and binds to LC3 on the autophagosome membrane. This interaction ultimately triggers mitophagy. In the ischemic state, PINK1 accumulates on the depolarized mitochondrial outer membrane and recruits activated Parkin, leading to mitophagy activation. Additionally, in the IRI state, S616 phosphorylation activates Drp1, which is translocated to the mitochondrial membrane and triggers mitophagy.

HIF-1α-BNIP3-mediated mitophagy in tubular cells protects against renal IRI [33]. Mechanistically, HIF-1α directly increases the transcription and expression of BNIP3 in IRI-AKI mice [33]. BNIP3 can bind to LC3 protein, and then connect autophagy vesicles to targeted mitochondria, directly induce mitophagy, while reducing the production of reactive oxygen species and improving renal IRI (Fig. 4) [32, 65]. Additionally, the PINK1-PRKN/PARK2 mitophagy pathway can be activated to protect against renal IRI [15]. This is due to ischemia-induced mitochondrial depolarization, during which Pink1 accumulates on the mitochondrial outer membrane and recruits Parkin from the cytoplasm. This activation of Parkin’s ubiquitin ligase activity triggers mitophagy, leading to the elimination of damaged mitochondria (Fig. 4) [66, 67]. After renal IRI, Drp1 is activated by S616 phosphorylation and translocated onto the mitochondrial membrane [34]. Subsequently, the Drp1-dependent mitophagy pathway can be activated, and mitophagic clearance of damaged mitochondria protects cells from IRI-induced apoptosis, thereby protecting the kidney (Fig. 4) [34]. Furthermore, ischemic preconditioning driven FUN14 domain protein 1 (Fundc1)-dependent mitophagy is activated by post-transcriptional phosphorylation of Ser17, which subsequently regulates mitochondrial fission to resist AKI, thereby ameliorating kidney injury [68]. Notably, Jin et al. found that UMI-77, a complex that induces mitophagy, attenuates renal TEC damage by enhancing mitophagy, downregulating the TGF-β/Smad signaling pathway, and subsequently inhibiting the epithelial-to-stromal transition phenotype, ultimately preventing renal fibrosis [69]. These findings suggest that targeting mitophagy may ameliorate kidney injury.

### Reducing ROS levels in renal tissue in ischemic AKI

Mitochondria are both the source and target of ROS in cells. Oxidative damage to mitochondria during reperfusion can further trigger or aggravate the induction of the cell death pathway, autophagy, and activation of the inflammasome, thus damaging renal function. Han et al. reported that mitochondrial NADP^+^-dependent isocitrate dehydrogenase (IDH2) deficiency exacerbates mitochondrial and cell damage following kidney IRI. Therefore, IDH2 is an important mitochondrial antioxidant enzyme that provides NADPH to promote ROS clearance and protects cells from IRI [70]. Bax inhibitor-1 (BI1), an evolutionarily conserved inhibitor of apoptosis, mediates the activation of extracellular signal-regulated kinase 1/2, leading to the inhibition of mitochondria-mediated ROS production. Wang et al. indicated that BI1 levels in the urine and plasma decrease in patients with AKI, and its expression inversely correlates with renal function. Mechanistically, BI1 interacts with anti-proliferative protein 2 (PHB2) to facilitate the translocation of cytoplasmic PHB2 into mitochondria with the assistance of TIM23. Once localized to mitochondria, PHB2 maintains mitochondrial morphology and function, while also inhibiting mitochondrial apoptosis. In addition, BI1 reconstitution in a murine AKI model reduced mitochondrial oxidative stress, inhibited excessive mitochondrial fission, improved mitophagy, and suppressed mitochondrial apoptosis [71]. Therefore, ROS production in the mitochondria can be inhibited by upregulating BI1 to improve kidney injury.

Mitoquinone (MitoQ), a mitochondria-targeted antioxidant that inhibits mitochondrial ROS production, accumulates in cells driven by the positive charge of the plasma membrane and further accumulates within mitochondria driven by the positive charge of the mitochondrial membrane. In the mitochondrial matrix, MitoQ is reduced to its active antioxidant form ubiquinol by the respiratory chain, thereby preventing oxidative damage such as lipid peroxidation. Antioxidant activity generates the ubiquinone form, which is subsequently recycled to ubiquinol by the respiratory chain, continuing its antioxidant effect. Dare et al. also found that MitoQ decreases mitochondrial oxidative damage, thus protecting against renal IRI in a mouse model [72].

Thus, treatment strategies that promote ROS clearance, inhibit ROS production, and protect mitochondria from oxidative damage may ameliorate renal IRI. BI1, the mitochondrial antioxidant enzyme IDH, and the mitochondria-targeted antioxidant MitoQ have been shown to reduce mitochondrial ROS levels and protect mitochondria from oxidative damage, thereby improving renal IRI.

### Targeting disrupted mitochondrial fusion and fission

Proximal tubule-specific deletion of Drp1 prevented renal IRI-induced kidney injury, inflammation, and programmed cell death observed in wild-type mice, and promoted epithelial recovery [73]. Wang et al. found that Mdivi-1 inhibition of Drp1-mediated mitochondrial fission alleviates renal interstitial fibrosis induced by unilateral ureteral obstruction [74]. Qi et al. designed a new selective peptide inhibitor P110 that inhibits Drp 1/Fis 1 interactions, thereby inhibiting mitochondrial breakage and ROS production [75, 76]. The above-mentioned inhibitors of genes Mdivi-1 and P110 can inhibit mitochondrial division to improve the function of tissues and organs such as the kidney.

Mfn2 protects against renal tubular apoptosis during ischemic AKI [77]. Thymoquinone, a strong free radical and superoxide scavenger, mitigates diclofenac-induced mitochondrial viability and apoptosis by upregulating Mfn2 expression, thereby improving renal function [78, 79].

Therefore, the regulation of key proteins involved in mitochondrial fusion and fission can inhibit apoptosis and ameliorate kidney injury.

### Other therapeutic approaches targeting mitochondria in AKI

The innate immune system has been implicated in AKI and CKD. Damaged mitochondria release noxious molecules, such as ROS, mtDNA, and cardiolipin, which can cause NLRP3 inflammasome activation, and the upregulation of IL-18 and IL-1b. Mitochondrial damage persists long after ischemia and sustains chronic inflammasome activation. However, using a mitoprotective agent SS-31 following ischemia preserves mitochondrial integrity, ameliorates expression levels of all inflammatory markers, restores glomerular capillaries and podocyte structure, and arrests glomerulosclerosis and interstitial fibrosis with the normalization of IL-18 and IL-1b expression. In the rat model of IRI, SS-31 treatment protected mitochondrial structure and respiration during early reperfusion, accelerated ATP recovery, reduced renal tubular cell necrosis, and reduced IR-mediated oxidative stress [80, 81].

Tan et al. suggested that fibroblast growth factor (FGF2) substantially ameliorates renal IRI by mitigating several mitochondrial damage parameters, including the pro-apoptotic alteration of Bcl2/Bax expression, caspase-3 activation, loss of mitochondrial membrane potential, and mitochondrial ATP-dependent potassium channel integrity. IRI alone results in mild activation of FGFR, whereas FGF2 treatment results in a more robust receptor activation. Notably, post-IRI administration of FGF2 also provides robust protection against IRI by reducing cell apoptosis, inhibiting the release of damage-associated molecular pattern molecule HMBG1, and activation of its downstream inflammatory cytokines, such as IL-1a, IL-6, and TNFα [82].

These findings suggest that mitochondrial protection is a novel therapeutic approach to arrest the progression of AKI to CKD. In addition, agents that open the mitochondrial ATP-dependent potassium channel are effective in preventing renal injury via the inhibition of mitochondrial DNA damage [83].

The replacement of damaged mitochondria also restores mitochondrial homeostasis. Jabbari et al. injected normal mitochondria isolated from healthy muscle cells into injured kidney cells via renal arteries in an animal model of AKI. Mitochondrial transplantation can prevent renal tubule cell death, restore renal function, improve renal injury, enhance renal tubule regeneration potential, and reduce IRI-induced apoptosis [84]. Although further research, including clinical trials, is urgently needed, mitochondrial transplantation offers a novel therapeutic strategy for AKI.

## Mitochondrial-related biomarkers for ischemia-induced AKI

Previous studies have shown a time-dependent loss of renal cortical mitochondrial proteins including ATP synthase subunit b (ATPSb) following IRI-AKI, indicating renal mitochondrial dysfunction [49, 85, 86]. Moreover, urinary full-length ATPSb has been identified as a specific biomarker for renal mitochondrial dysfunction [87]. In patients who have undergone surgery and developed postoperative AKI, urinary ATPSb levels have been found to be elevated. Similarly, in the IRI-AKI mouse model, urinary full-length ATPSb levels increased with moderate and severe kidney injury. This increase preceded the elevation of other markers of kidney injury [87]. These findings suggest that urinary full-length ATPSb may serve as a sensitive and specific translational biomarker for renal mitochondrial dysfunction in IRI-AKI.

Elevated mtDNA levels may serve as surrogate biomarkers of mitochondrial dysfunction, kidney damage, and the progression and prognosis of kidney diseases [88]. Following ischemic AKI, considerable mitochondrial damage occurs in renal tubular cells and podocytes, resulting in the release of mtDNA into the extracellular space, and subsequently, in systemic circulation. Thereafter, free mtDNA is detected in the plasma and used as a biomarker of kidney injury. Simultaneously, damaged mtDNA from renal parenchymal cells leaks into urine because of kidney injury, and elevated urinary mitochondrial DNA levels can be used as a surrogate biomarker for kidney injury [89]. Therefore, the detection of elevated mtDNA levels in plasma and urine is significant to advance our understanding of IRI pathogenesis in AKI and the role of targeting mtDNA in IRI. The search for mitochondrial biomarkers of ischaemic-induced AKI is a new field and direction, and only a few studies have been conducted so far, which deserves our attention and further study.

Pannexin 1 (PANX1) is a regulator of ATP release [90, 91]. In an oxidative environment, PANX1 activates autophagy signaling by increasing extracellular ATP efflux and engaging purinergic P2X and P2Y signaling pathways [92]. Furthermore, P2Y receptors promote the rapamycin target protein signaling pathway and regulate mitochondrial activity [93]. Therefore, PANX1 activation leads to ATP release, which stimulates P2Y receptors, resulting in activation of mTOR signaling and ultimately inhibiting mitophagy [94]. Research by Su et al. has shown that PANX1 levels are elevated in the serum of patients who develop AKI following cardiac surgery [94]. These findings suggest that PANX1 may serve as a potential mitochondria-related biomarker for the diagnosis of IRI-AKI.

There is a pressing need for early, specific, and non-invasive biomarkers of mitochondrial dysfunction in IRI-AKI. The identification and validation of such markers could provide valuable insights into the underlying mechanisms of renal mitochondrial dysfunction in AKI and lead to the development of novel therapeutic targets.

## Conclusion and perspectives

Severe tubular injury is a characteristic of AKI. Extensive necrosis and apoptosis of TECs is observed, especially in the proximal tubules. Mitochondria in the proximal tubular epithelium play a key role in the pathophysiology of IRI-AKI. The morphology and function of mitochondria change considerably in AKI. The most obvious morphological changes are mitochondrial swelling and fragmentation, disruption of membrane integrity, and mitochondrial ridge fractures or deletions. Subsequent mitochondrial dysfunction manifested as reduced ATP production, elevated ROS levels, and the release of pro-apoptotic factors, further damages the kidneys.

In this paper, several targets of mitochondrial-targeted therapy are discussed. In targeted mitochondrial energy metabolism therapy, renal IRI can be improved by modulating CPT1 to increase PPARα-regulated FAO levels [51]. However, in the mouse kidney fibrosis model, it can still maintain mitochondrial homeostasis by affecting CPT1A, thereby improving kidney injury [52]. Previous studies have shown that AICAR has a protective effect against ischemia-reperfusion injury in the heart and liver, which further supports the great potential of AICAR in the treatment of IRI-AKI [64]. In addition, regulating key enzymes (such as PDH, CS, AKGDH, etc.) in the process of TCA and related complexes (such as complex I, complex II, etc.) in the process of oxidative phosphorylation can preserve or restore mitochondrial energy metabolism in IRI-AKI, thereby improving kidney injury [53–55]. Therapeutic measures targeting mitochondrial energy metabolism have been more studied and confirmed in other organ or disease models, which has a very positive significance in guiding the treatment of IRI-AKI.

In targeted mitophagy therapy, HIF-1α-BNIP3 and PINK-PRKN/PARK2 eliminate damaged mitochondria and reduce ROS levels by directly inducing or activating mitophagy, thereby reducing mitochondrial damage and achieving the purpose of protecting renal function [15, 33]. Although Drp1 and Fundc1 have also been confirmed in IRI-AKI to activate mitophagy and play a protective role in kidney injury, the specific molecular mechanisms are not fully understood [34, 68]. The protective effect of UMI-77 on the kidney by activating mitophagy in the renal fibrosis model also enables us to make a reasonable hypothesis that UMI-77 can activate mitophagy to improve kidney injury in IRI-AKI.

ROS is a key factor affecting mitochondrial damage in AKI, and targeting ROS is the main direction of AKI treatment. In addition, there are many effective antioxidant drugs (such as n-acetylcysteine, MitoQ, etc.) that have been shown to play a beneficial role in the ischemia/reperfusion process of the liver, heart, and kidney [95–100].

For IRI-AKI, several key targets of IDH2, BI1 and MitoQ are discussed in this paper. In particular, the therapeutic role of MitoQ in IRI-AKI has been well established, and studies have shown that MitoQ can be used as a potential drug for post-resuscitation myocardial infarction [101]. These studies fully demonstrate that MitoQ plays a positive therapeutic role in IRI-AKI. This also suggests that the development of more mitochondria-targeting antioxidants will be of great significance for the future treatment of IRI-AKI. In targeting mitochondrial fusion and fission, we focused on the therapeutic effect of Mdivi-1 on IRI-AKI. Mdivi-1 also plays a protective role in ischemic reperfusion injury in the brain and heart muscle [102, 103]. Other targets for mitochondrial therapy (such as SS-31 and FGF2) are also very meaningful and worth exploring. We believe that these are the hot spots and new directions of IRI-AKI therapy in the future.

Studies have confirmed multiple therapeutic targets for AKI; however, no targeted drugs are currently approved for the treatment of AKI in clinical practice. SIRT5, MitoQ, and BNIP3 have shown promising potential for treating mitochondrial damage and kidney disease in clinical trials concerning certain kidney injuries and diseases. In the future, we can regulate the levels of peroxisome and mitochondrial fatty acid oxidation in proximal tubule epithelial cells by inhibiting SIRT5, thereby improving AKI. Moreover, the development of BNIP3 activators to reduce ROS production will make it possible to alleviate AKI symptoms. In addition, we can also develop reducing agents for MitoQ to activate it to prevent oxidative damage and improve AKI. Therefore, targeted AKI therapy remains an active area of research. With the progress in human clinical trials on the efficacy and safety of mitochondria-targeted therapy approaches, we hope that mitochondrial therapeutic targets become a strategy to improve kidney injury and disease in the future.

## Data Availability

All data that support the findings of this study are available from the corresponding author upon reasonable request.
